# Occurrence, Accumulation, and Impacts of Environmental Pollutants in Aquatic Systems

**DOI:** 10.3390/toxics13110994

**Published:** 2025-11-19

**Authors:** Hongbin Lu, Zhuowei Zhang, Fanhao Song

**Affiliations:** 1 State Key Laboratory of Coal Conversion, Institute of Coal Chemistry, Chinese Academy of Sciences, Taiyuan 030001, China; luhongbin@sxicc.ac.cn; 2 Hebei Engineering Research Center for Sewage Treatment and Resource Utilization, School of Water Conservancy and Hydroelectric Power, Hebei University of Engineering, Handan 056038, China; 3 Hebei Technology Innovation Center of Water Pollution Control and Water Ecological Remediation, School of Water Conservancy and Hydroelectric Power, Hebei University of Engineering, Handan 056038, China; 4 State Key Laboratory of Environmental Criteria and Risk Assessment, Chinese Research Academy of Environmental Sciences, Beijing 100012, China

## 1. Introduction

In response to the growing concerns of environmental pollution and its ecological impacts, this collection of research focuses on the monitoring, behavior, and remediation of various contaminants in aquatic systems. The studies address a range of toxic agents—including heavy metals (e.g., As, Cd, Cr, Cu, Pb) [1], endocrine disruptors like 17β-estradiol [2], and petroleum hydrocarbons [3]—originating from industrial, agricultural, and urban activities. These pollutants enter water bodies, accumulate in aquatic organisms, alter microbial community structures [4], and influence human health and safety. There is an increasing emphasis on developing sensitive, rapid, and eco-friendly monitoring tools, such as electroactive microorganism-based biosensors [5], and on understanding the underlying mechanisms of toxicity and pollutant migration. Concurrently, bioremediation and phytoremediation strategies using native plants [6] and microorganisms [7] are being explored as sustainable solutions to mitigate contamination.

Following a thorough review process, the selected articles collectively highlight innovative approaches in environmental toxicology and remediation. Some studies present novel biosensing techniques using microbial fuel cells for real-time toxicity warning systems [8], while others investigate the use of marine photoautotrophs—such as mangroves, seagrasses, and seaweeds—for effective pollutant removal [9]. Several papers analyze the bioaccumulation of trace metals in marine species and assess ecological risks using bacterial community biomarkers. Additionally, research on the mitigation of lead toxicity in fish using plant-based treatments demonstrates practical bioremediation applications. Together, these contributions represent significant advances in the detection, behavior analysis, and biological remediation of environmental pollutants, offering valuable tools and insights for coastal, aquatic, and wastewater management.

## 2. An Overview of Published Articles

The review article by Xing et al., “*Research on the Application and Mechanisms of Electroactive Microorganisms in Toxicants Monitoring: A Review*” (contribution 1), presents the principles, applications, and influencing factors of using electroactive microorganisms in microbial fuel cells (MFCs) for toxicity monitoring. The core principle is that electroactive microorganisms generate electrical signals, which immediately decrease when their activity is inhibited by toxicants. Common MFC configurations include dual-chamber and single-chamber reactors, and their performance can be enhanced through electrode material modification [10]. The voltage inhibition rate is the most commonly used indicator for toxicity assessment [11]. The review identifies that factors such as flow rate, culture time, substrate concentration, and sodium chloride concentration significantly affect the sensitivity and stability of the biosensor. The mechanisms of toxicity involve the inhibition of microbial energy metabolism, key enzymes, and the induction of oxidative stress, which disrupt extracellular electron transfer. Finally, compared to traditional anaerobic toxicity assays (e.g., based on methane production) or luminescent bacteria tests, MFC-based biosensors offer the advantages of rapid response and potential for online monitoring, making them a promising tool for wastewater toxicity early warning systems.

The article by Gao et al., entitled “*Effects of 17β-Estradiol Pollution on Microbial Communities and Methane Emissions in Aerobic Water Bodies*” (contribution 2), investigates the impact of the endocrine-disrupting compound 17β-estradiol (E2) on microbial communities and greenhouse gas emissions in laboratory-simulated aerobic aquatic systems. Experiments were conducted with E2 concentrations of 0 ng/L (control), 100 ng/L (low), and 10,000 ng/L (high). The results showed that E2 contamination stimulated short-term methane (CH_4_) emissions, particularly within the first two days. Using 16S rRNA sequencing, the study found that E2 increased the stochasticity of bacterial and archaeal community assembly and weakened microbial interactions. Specifically, E2 pollution significantly decreased the relative abundance of *Proteobacteria* (which includes methanotrophs) [12] and increased the relative abundance of *Planctomycetota* (which may contribute to aerobic methane production) [13]. Functional prediction indicated an increase in the methanogenesis group and a decrease in the methanotrophy group. The study concluded that E2 promotes (CH_4_) emissions through three pathways, namely stimulating methanogens like Methanoregula in anoxic microsites, boosting *Planctomycetota* capable of methylphosphonate utilization, and inhibiting methanotrophic bacteria.

The article by Li et al., entitled “*Bioaccumulation of Arsenic, Cadmium, Chromium, Cobalt, Copper, and Zinc in Uroteuthis edulis from the East China Sea*” (contribution 3), analyzes the concentration and distribution of six trace elements (TEs) in different tissues of the squid Uroteuthis edulis. The tissues analyzed were the mantle, digestive gland, gonad, and gill. The study found significant differences in TE concentrations among tissues. The digestive gland accumulated the highest concentrations of Cu, Zn, and Cd, while the gill showed the highest levels of Cr and Co. Arsenic (As) was relatively evenly distributed across all tissues. In terms of the total body burden of TEs, the mantle contributed the highest proportion due to its large mass. Correlation analysis revealed significant positive correlations among Cu, Co, and Cd in certain tissues, possibly related to metallothionein binding. Furthermore, the study compared TE concentrations before and after gonadal maturation. Significant increases in the concentrations of most TEs (except Zn) were observed in the digestive gland of mature individuals, and significant increases in Cr, Cu, and As were found in the gonads, suggesting potential maternal transfer of these elements to offspring.

The article by Roda F. Al-Thani and Bassam T. Yasseen, entitled “*Methods Using Marine Aquatic Photoautotrophs along the Qatari Coastline to Remediate Oil and Gas Industrial Water*” (contribution 4), presents the potential of native marine organisms for remediating pollutants from oil and gas wastewater [14]. The study focused on marine photoautotrophs including the mangrove Avicennia marina, seagrasses (*Halodule uninervis*, *Halophila ovalis*, *Thalassia hemprichii*), and various species of green, brown, and red seaweeds. These organisms and their associated microorganisms were found to remove heavy metals and degrade petroleum hydrocarbons through mechanisms such as phytoextraction, phytostabilization, and phycoremediation [15]. The mangrove A. marina proved efficient in accumulating heavy metals such as Co, Cr, Cu, Fe, Ni, and Zn. Seagrasses were identified as promising candidates for phytoremediation or as bioindicators. Seaweeds demonstrated a high capacity for the biosorption and bioaccumulation of common heavy metals like As, Cd, and Hg [16]. The remediation process was enhanced by the synergistic relationship between plants and their associated microorganisms, where root exudates stimulated microbial activity to degrade organic pollutants.

The article by Li et al., entitled “*Characteristics and Mechanism of Hematite Dissolution and Release on Arsenic Migration in Heterogeneous Materials*” (contribution 5), presents the impact of hematite dissolution on the migration and adsorption of arsenic in a heterogeneous aquifer system. A stratified sand column embedded with a hematite lens at the coarse-to-medium sand interface was designed to simulate groundwater flow. The medium structure significantly influenced arsenic migration, where clay layers directed the lateral migration of arsenic, leading to concentrations in deeper layers up to seven times greater than those on the surface. Solid-phase extraction revealed that arsenic was primarily adsorbed on quartz sand surfaces in a specifically adsorbed state (F2) [17] and bound to amorphous iron–aluminum oxides (F3). Monitoring of aqueous iron (Fe(aq)) showed a rapid increase to a maximum on day 15, followed by a gradual decline, indicating that hematite dissolution was not continuous. The released Fe(aq) subsequently contributed to the formation of fresh iron–aluminum oxides that adsorbed As(V), thereby reducing its concentration and influencing its spatial distribution within the sand column.

The article by Xia et al., entitled “*Using Sediment Bacterial Communities to Predict Trace Metal Pollution Risk in Coastal Environment Management: Feasibility, Reliability, and Practicalility*” (contribution 6), presents the distribution and accumulation risk of trace metals (Al, As, Cr, Cu, Fe, Mn, Ni, Sr, Zn) in a tidal gate-controlled coastal river. The study area was divided into the catchment area (CA), estuarine area (EA), and offshore area (OA). The enrichment factor and geoaccumulation index identified As and Cr as the key pollutants, reaching slight-to-moderate pollution levels. The Nemero pollution index was highest in the EA (14.93), indicating a significant pollution risk near the tidal gates. Although Fe and Mn dynamics could partially explain trace metal behavior, they showed no linear relationships with toxic metals. Interestingly, the metabolic abundance of sediment bacterial communities showed strong correlations with various trace metals [18]. These results indicate that bacterial community characteristics can serve as effective biomarkers for assessing trace metal pollution and offer a practical tool for coastal environmental management.

The article by Nimai Chandra Saha et al., entitled “*Toxic Effects of Lead Exposure on Freshwater Climbing Perch, Anabas testudineus, and Bioremediation Using Ocimum sanctum Leaf Powder*” (contribution 7), presents the toxic effects of lead (Pb) on fish and its remediation. The 96 h LC50 value of lead for Anabas testudineus was determined to be 1.08 mg/L using static replacement bioassay. Chronic exposure to sublethal concentrations (10% and 20% of LC50) significantly lowered growth parameters (hepatosomatic index, specific growth rate), hematological biomarkers (RBC, hemoglobin), and increased serum enzyme levels (AST, ALT). Scanning electron microscopy revealed abnormal shapes and surfaces of erythrocytes in exposed fish. The leaf powder of Ocimum sanctum was administered with fish food to mitigate lead toxicity. The results showed that fish treated with the mixture of lead and Ocimum sanctum leaf powder exhibited significant recovery in growth, as well as hematological and biochemical parameters compared to those exposed to lead alone, indicating the remedial role of Ocimum sanctum against lead toxicity.

The article by Porttape Jendanklang et al., entitled “*The Contamination of Microplastic Debris in Blue Swimming Crab Portunus pelagicus from Artisanal Fisheries in the Eastern Gulf of Thailand*” (contribution 8), presents the microplastic (MP) contamination in crabs collected from the coast of Rayong province. The crab samples were collected from four sites in January, April, and August 2024, representing different monsoon seasons. MPs were examined in both external and internal body parts. The overall detection rate of MPs was 72.2% internally and 62.5% externally. The gut was the most contaminated tissue, followed by the gills, while no MPs were found in the hepatopancreas or muscle. MP abundance showed significant seasonal variation, with the highest level in August. Fibers were the dominant shape, blue was the most common color, and the primary polymers identified were polyethylene terephthalate glycol (PETG), nylon, and polypropylene. The study indicates that household laundry fibers and damaged fishing gear are major sources of MP pollution. It is concluded that improving waste management and developing more durable fishing gear are crucial for mitigating this contamination.

## Data Availability

No new data were created or analyzed in this study. Data sharing is not applicable to this article.

## References

[1] Ali M.M., Awan F., Jamil M.U., Ijaz M., Ullah A., Shehzad W. (2025). Genotoxic and oxidative stress assessment of heavy metal contaminated soil and water in human blood and bovine milk samples from the different industrial zones of Punjab, Pakistan. J. Trace Elem. Med. Biol..

[2] Alahadeb J.I. (2022). Effective biodeterioration of a common endocrine disruptor 17β-estradiol using mixed microbial cultures isolated from waste water. Environ. Res..

[3] Wang M., Wang C., Li Y. (2017). Petroleum hydrocarbons in a water-sediment system from Yellow River estuary and adjacent coastal area, China: Distribution pattern, risk assessment and sources. Mar. Pollut. Bull..

[4] Hong Y., Li D., Xie C., Zheng X., Yin J., Li Z., Zhang K., Jiao Y., Wang B., Hu Y. (2022). Combined apatite, biochar, and organic fertilizer application for heavy metal co-contaminated soil remediation reduces heavy metal transport and alters soil microbial community structure. Sci. Total Environ..

[5] Xi H., Wang H., Li Y., Long X., Li X., Sun Y., Wang W. (2025). Advancing Shewanella-based whole-cell biosensors: A comprehensive review on heavy metal detection. Colloids Surf. B Biointerfaces.

[6] Liu N., Zhao J., Du J., Hou C., Zhou X., Chen J., Zhang Y. (2024). Non-phytoremediation and phytoremediation technologies of integrated remediation for water and soil heavy metal pollution: A comprehensive review. Sci. Total Environ..

[7] Taharia M., Dey D., Das K., Sukul U., Chen J.-S., Banerjee P., Dey G., Sharma R.K., Lin P.-Y., Chen C.-Y. (2024). Microbial induced carbonate precipitation for remediation of heavy metals, ions and radioactive elements: A comprehensive exploration of prospective applications in water and soil treatment. Ecotoxicol. Environ. Saf..

[8] Safwat S.M., Khaled A., Elawwad A., Matta M.E. (2023). Dual-chamber microbial fuel cells as biosensors for the toxicity detection of benzene, phenol, chromium, and copper in wastewater: Applicability investigation, effect of various catholyte solutions, and life cycle assessment. Process Saf. Environ. Prot..

[9] Talaiekhozani A., Rezania S. (2017). Application of photosynthetic bacteria for removal of heavy metals, macro-pollutants and dye from wastewater: A review. J. Water Process Eng..

[10] Zhang Q., Tan B., Li H., An F. (2026). Nitrogen-doped and catalytically modified electrodes in campus-sourced electrogenic microbe-driven MFCs for enhanced copper ion adsorption. Bioelectrochemistry.

[11] Lee C., Park Y.H., Kang S.W. (2025). Nanoporous Cellulose Acetate-Coated Polypropylene Membranes for Microbial Fuel Cells: Enhanced Long-Term Stability, Fouling Resistance, and Reverse Voltage Suppression. Biomacromolecules.

[12] Li J., Liu R., Liu X., Yang Q., Zhang S. (2025). Organic matter removal and CH4 production performance recoveries and microbial community changes in upflow anaerobic biofilter after long term starvation. J. Environ. Sci..

[13] Klimek D., Herold M., Calusinska M. (2024). Comparative genomic analysis of Planctomycetota potential for polysaccharide degradation identifies biotechnologically relevant microbes. BMC Genom..

[14] Kianfar E. (2025). Current situation and future outlook petroleum hydrocarbons in marine systems: A review. Environ. Technol. Innov..

[15] Cozma P., Roșca M., Minuț M., Gavrilescu M. (2025). Phytoremediation: A sustainable and promising bio-based approach to heavy metal pollution management. Sci. Total Environ..

[16] Greeshma K., Kim H.-S., Ramanan R. (2022). The emerging potential of natural and synthetic algae-based microbiomes for heavy metal removal and recovery from wastewaters. Environ. Res..

[17] Guo H., Stüben D., Berner Z. (2007). Arsenic removal from water using natural iron mineral–quartz sand columns. Sci. Total Environ..

[18] Bo H., Li Z., Wang H., Zhang H., Xu R., Xue D., Li H., Wang W., Zhang W., Zhang Q. (2025). Long-term exposure to fly ash leachate enhances the bioavailability of potentially toxic metals and decreases bacterial community diversity in sediments. J. Environ. Manag..

